# Low dose of mycophenolate mofetil is enough in desensitized kidney transplantation using rituximab

**DOI:** 10.1186/s12882-015-0201-7

**Published:** 2015-12-04

**Authors:** Chung Hee Baek, Hyosang Kim, Hoon Yu, Eunhye Shin, Hyungjin Cho, Won Seok Yang, Duck Jong Han, Su-Kil Park

**Affiliations:** Division of Nephrology, Department of Internal Medicine, Asan Medical Center, University of Ulsan College of Medicine, Seoul, Republic of Korea; Department of Surgery, Asan Medical Center, University of Ulsan College of Medicine, Seoul, Republic of Korea

**Keywords:** Rituximab, Kidney transplantation, Immunosuppression

## Abstract

**Background:**

Rituximab is widely used in kidney transplantation. However, it is not clear whether the conventional doses of maintenance immunosuppressant in rituximab-treated kidney transplantation (KT) are appropriate. In our previous study, decreasing mycophenolate mofetil (MMF) dose due to infection did not increase the incidence of rejection or graft failure. Based on these experiences, we developed a new protocol with a lower dose of MMF and studied its clinical outcomes in rituximab-treated KT.

**Methods:**

We enrolled all patients who underwent ABO-incompatible or human leukocyte antigen (HLA)-sensitized living donor KT with the new immunosuppressant protocol after preconditioning with rituximab, but without splenectomy from November 2011 to May 2013. Seventy-two patients (group 1) were consecutively enrolled in this study and followed until November 2013. Patients from our previous study served as control groups. Sixty-seven patients received KT using rituximab with a conventional dose of MMF (group 2), and 87 patients received ABO compatible KT without need for rituximab (group 3). Clinical outcomes, including rejection, infection, and graft survival, were compared between the groups. The *χ*^2^ test and Fisher’s exact test were used for categorical variables, the Student’s *t*-test and Mann-Whitney *U* test were used for continuous variables, and a log-rank test was used for mortality analysis.

**Results:**

Doses of postoperative MMF (g/day) were lower in group 1 than in the other groups (1.03 ± 0.19, 1.48 ± 0.34 and 1.48 ± 0.32 g/day at 1 week, *p* < 0.001). Infectious complications occurred more often in groups with conventional MMF doses (group 2 and 3) than in group 1 (16.7 vs. 37.3 %, *p* = 0.007 and 16.7 vs. 34.5 %, *p* = 0.012, respectively). Notably, group 1 showed a lower incidence of cytomegalovirus infection than group 2. However, reduction in MMF dose did not increase the incidence of acute rejection (4.2, 4.5 and 10.3 %). Only one graft failure occurred in group 2 due to vessel kinking after operation. There were no significant differences in the incidence of malignancy and mortality between groups.

**Conclusions:**

A low MMF dose reduces infection without increasing rejection or graft loss and it may be appropriate to reduce the dose of MMF for rituximab-treated KT patients.

## Background

Rituximab, a chimeric anti-CD20 monoclonal antibody, is widely used in desensitization protocols these days. It was first used for ABO incompatible (ABOi) kidney transplantation (KT) combined with plasmapheresis and splenectomy in 2002 [1]. ABOi KT using rituximab instead of splenectomy as a desensitization protocol was reported within the following year [2]. Transplantations in HLA sensitized patients have recently been performed and ABOi/HLA-sensitized KT using rituximab has led to successful results [3–5].

Some retrospective studies have reported that rituximab is associated with an increased risk of infectious complications [6, 7]. However, a randomized, double-blind, placebo-controlled study showed a trend toward fewer and milder rejections during the first 6 months in the rituximab group with no increase in infectious complications [5]. In our previous study [8], serious infectious complications occurred more often in the rituximab-treated KT. Therefore, the doses of mycophenolate mofetil (MMF) had to be decreased in the rituximab group to avoid or to cure serious infection. However, acute rejection was not observed with the reduced doses of MMF. After these experiences, we changed our immunosuppressant protocol for ABOi KT and HLA sensitized KT to a lower dose of MMF (1.0 g/day after postoperative week 1 in advance), and collected data prospectively without omitting any patients. So far, no guidelines have been suggested for a safe and effective maintenance immunosuppressive regimen in rituximab- and IL-2-receptor antibody-treated KT.

In this study, we evaluated our protocol of low dose of MMF in rituximab-treated KT, focusing on its associations with reduced infectious complications without increased incidence of acute rejection.

## Methods

### Patients

We enrolled all patients who underwent ABOi or HLA-sensitized, living-donor KT with the new immunosuppressant protocol after preconditioning with rituximab but without splenectomy (group 1) at the Asan Medical Center, a tertiary referral teaching hospital in Seoul, Korea, from November 2011 to May 2013. Seventy-two consecutive patients were enrolled for this study and followed until November 2013. No patients were lost to follow-up during the study period except in cases of death. Patients received tacrolimus, MMF, and corticosteroids as maintenance immunosuppressants according to the new protocol. Patients enrolled in our previous study [8] served as controls. Of these, 67 patients received desensitized KT using rituximab (group 2) and 87 patients received ABO compatible KT (group 3) between January 2009 and May 2011. They all received conventional doses of maintenance immunosuppressants. This study was approved by Asan Medical Center Institutional Review Board (2014–0724), and informed consent was obtained from all participants.

### Immunosuppression protocol

**Fig. 1:**
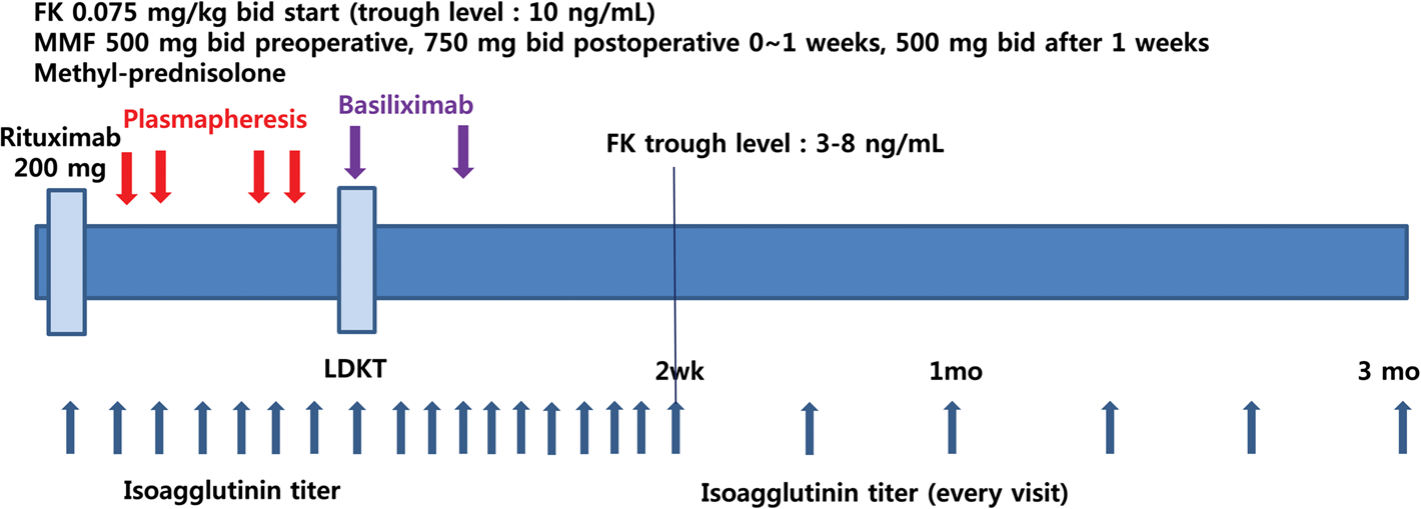
New immuonosuppressive protocol. Tacrolimus, mycophenolate mofetil (MMF) and steroids are used in addition to rituximab and started 7–10 days before the operation. Tacrolimus is started at a dose of 0.075 mg/kg bid, and the target drug trough level is 10 ng/mL for the first 2 postoperative weeks and 3–8 ng/mL after 2 weeks. MMF is started at a dose of 500 mg bid before the operation, and 750 mg bid is used for the first postoperative week. After 1 week, MMF dose is reduced to 500 mg bid

The new immunosuppressant protocol for rituximab-treated renal transplantation is summarized in Fig. 1. Tacrolimus, MMF, and steroids were used in addition to rituximab and started 7–10 days before the operation. Tacrolimus was started at a dose of 0.075 mg/kg bid, and the target drug trough level was 10 ng/mL for postoperative weeks 1–2 and 3–8 ng/mL thereafter. The starting dose of MMF was 500 mg bid and elevated to 750 mg bid from day 0 to day 7 postoperatively. After 1 week, the dose of MMF was reduced to 500 mg bid. In groups 2 and 3, the conventional dose of 1.5 g/day of MMF was used for at least 1 month postoperatively. Rituximab was administered 7–10 days before the operation. We used 200 mg/body rituximab for ABOi living donor transplantation, while 500 mg/body rituximab was used in patients with a positive crossmatch on T-cell flow cytometry. Basiliximab, an anti-CD25 monoclonal antibody, was administered to all patients on the day of the operation and on postoperative day 4. No patient received splenectomy or intravenous immunoglobulin (IVIG) injection. Several rounds of plasmapheresis were performed until the ABO isoagglutinin titer was reduced to less than 1:8 or T-cell flow cytometry (COBE® Spectra, CaridianBCT, USA) was negative.

### Diagnosis of rejection

Acute rejection was diagnosed on the basis of the Banff criteria, and C4d staining was performed in all specimens. Protocol biopsies were not routinely performed. Renal biopsies were performed to confirm rejection when clinically suspected by elevated serum creatinine.

### Definition of infection

Cytomegalovirus (CMV) infection was defined as positivity on a CMV antigenemia assay ≥50 cells/200,000 white blood cells and treated with ganciclovir. When plasma BK virus DNA was greater than 10,000 copies/ml, BK virus infection was diagnosed regardless of nephropathy. The CMV antigenemia assay and BK virus PCR were performed as previously reported, and the definition of infection was the same as the previous study [8]. CMV prophylaxis was not performed routinely because our nation-wide insurance system supports oral valganciclovir prophylaxis only for a case of donor’s CMV IgG is positive and recipient’s CMV IgG is negative. Instead, we checked CMV antigenemia routinely at 1, 2, 3, 4, 6, 8, 12, 16, 20 and 24 weeks after KT and performed preemptive therapy [9]. All patients received oral trimethoprim/sulfamethoxazole (80/400 mg) for pneumocystis pneumonia prophylaxis until 6 months post-operation.

### Statistical analysis

Statistical analyses were performed with SPSS 21 (IBM Corp., Armonk, NY, USA). Data are expressed as mean ± standard deviation or counts and percentages. For categorical variables, the *χ*^2^ test and Fisher’s exact test were used. Continuous variables were compared using the Student’s *t*-test or Mann-Whitney *U* test. Mortality rates were evaluated by the Kaplan-Meier method with a log-rank test. All statistical tests were two-tailed and *P* value <0.05 was considered significant.

## Results

### Baseline clinical characteristics

Seventy-two patients were enrolled in group 1 (reduced dose of MMF), and 67 and 87 patients were enrolled in groups 2 and 3 (conventional dose of MMF), respectively. The mean age was not significantly different between groups 1 and 3, but group 2 patients were significantly older than group 1 patients (40.92 ± 10.20 years vs. 44.88 ± 11.65 years, *p* = 0.034). In addition, the donors of group 1 were younger than the donors of group 3 (39.63 ± 10.95 vs. 43.00 ± 9.90 years, *p* = 0.043). There were more glomerulonephritis-caused cases of end stage renal disease in group 1. Most patients in all groups were positive for CMV IgG, and the CMV IgG serostatus of donor-recipient pairs was not different between groups. The mean follow-up time was 14.89 ± 6.01, 12.63 ± 7.59, and 14.05 ± 8.17 months in groups 1–3, respectively, without significant difference. Baseline characteristics are summarized in Table 1.

**Table 1:** Baseline clinical characteristics

	Low dose MMF	Conventional dose MMF		p1^a^	p2^b^
	Rituximab(+)	Rituximab(+)	Rituximab(−)		
	Group 1 (*n* = 72)	Group 2 (*n* = 67)	Group 3 (*n* = 87)		
Sex (male/female)	34/38	42/25	46/41	0.088	0.526
Age (years)	40.92 ± 10.20	44.88 ± 11.65	42.72 ± 10.39	0.034	0.273
Dialysis (hemo/peritoneal)	50/13	57/6	54/13	0.134	1.000
Dialysis duration (months)	24.95 ± 30.20	32.11 ± 33.47	31.04 ± 39.09	0.210	0.324
Etiology of ESRD				0.043	0.006
Diabetes mellitus	13	14	21		
Hypertension	11	12	23		
Glomerulonephritis	33	16	14		
Vesicoureteral reflux	0	4	1		
Polycystic kidney disease	3	5	5		
Unknown etiology	11	11	15		
Other causes	1	5	7		
Past medical history					
Diabetes mellitus	14	15	23	0.682	0.348
Hypertension	59	57	73	0.655	0.833
Hepatitis	4	6	2	0.522	0.411
Autoimmune disease	3	0	1	0.246	0.329
Malignancy history	1	3	0	0.352	0.453
Mean follow up time (months)	14.89 ± 6.01	12.63 ± 7.59	14.05 ± 8.17	0.053	0.455
ABO compatibility/T-flow				0.255	
ABO incompatible	47	36	-		
/T-flow(−)					
ABO compatible	14	21	-		
/T-flow(+)					
ABO incompatible	11	10	-		
/T-flow(+)					
Donor to recipient				0.159	
Compatible	14	21	87		
A → B	6	11	-		
B → A	9	8	-		
A/B → O	24	18	-		
AB → A/B/O	19	9	-		
HLA mismatch	3/7/13/14/10/19/6	4/0/7/20/7/18/11	4/2/12/25/14/19/11	0.065	0.335
(0/1/2/3/4/5/6)					
HLA class I mismatch	5/14/25/16/12	4/5/27/14/17	5/8/34/23/17	0.260	0.441
(0/1/2/3/4/)					
HLA class II mismatch	13/32/27	6/33/28	10/49/28	0.296	0.275
(0/1/2/)					
Donor sex (male/female)	39/33	34/33	46/41	0.735	0.875
Donor age (years)	39.63 ± 10.95	41.88 ± 11.65	43.00 ± 9.90	0.241	0.043
Donor’s relation with recipient				0.822	0.011
Parents	8	7	8		
Siblings	17	20	36		
Children	14	11	6		
Spouse	27	21	22		
Others	6	8	15		
CMV IgG serostatus				0.178	0.590
Donor+/Recipient+	70 (97.2 %)	65 (97.0 %)	86 (98.9 %)		
Donor+/Recipient-	0 (0.0 %)	2 (3.0 %)	0 (0 %)		
Donor-/Recipient+	2 (2.8 %)	0 (0.0 %)	1 (1.1 %)		

*Abbreviations*: *CMV* cytomegalovirus, *ESRD* end stage renal disease, *HLA* human leukocyte antigen, *MMF* mycophenolate mofetil

^a^group 1 vs. group 2

^b^group 1 vs. group 3

### Levels and doses of immunosuppressant

MMF doses were significantly lower in group 1 than in group 3 (Table 2). However, MMF doses in group 1 were significantly lower compared with group 2, predominantly in the early phase after transplantation, as MMF doses in group 2 had to be decreased due to infectious complications in many cases. Tacrolimus levels were not different between groups post-transplantation. The doses of methyl-prednisolone were lower in group 1 than in group 2. However, there was no difference between groups 1 and 3.

**Table 2:** Doses of immunosuppressants

	Low dose MMF	Conventional dose MMF		p1^a^	p2^b^
	Rituximab(+)	Rituximab(+)	Rituximab(−)		
	Group 1	Group 2	Group 3		
	(*n* = 72)	(*n* = 67)	(*n* = 87)		
Tacrolimus drug levels (ng/ml)					
Pre KT (72/67/87)	6.99 ± 2.57	9.56 ± 4.75	11.11 ± 6.58	<0.001	<0.001
After 1 month (72/65/87)	7.74 ± 3.53	9.17 ± 3.22	7.65 ± 2.89	0.015	0.847
After 3 months (71/55/86)	7.19 ± 2.79	7.64 ± 2.54	7.58 ± 2.76	0.349	0.380
After 6 months (71/48/71)	6.66 ± 2.50	7.11 ± 2.11	6.83 ± 2.19	0.304	0.664
After 1 year (49/39/48)	5.77 ± 1.81	6.57 ± 2.13	5.77 ± 2.16	0.060	0.987
After 2 years (6/4/15)	7.18 ± 1.28	5.48 ± 1.81	5.51 ± 3.16	0.116	0.228
Mycophenolate mofetil doses (g/day)					
Pre KT (72/67/87)	1.44 ± 0.15	1.51 ± 0.29	1.55 ± 0.17	0.071	<0.001
After 1 week (72/66/87)	1.03 ± 0.19	1.48 ± 0.34	1.48 ± 0.32	<0.001	<0.001
After 2 weeks (72/66/87)	1.00 ± 0.15	1.42 ± 0.39	1.48 ± 0.31	<0.001	<0.001
After 3 weeks (72/66/87)	1.00 ± 0.17	1.39 ± 0.41	1.46 ± 0.32	<0.001	<0.001
After 1 month (72/65/87)	0.95 ± 0.24	1.26 ± 0.42	1.40 ± 0.39	<0.001	<0.001
After 3 months (71/55/86)	0.93 ± 0.30	1.14 ± 0.51	1.35 ± 0.38	0.007	<0.001
After 6 months (71/48/71)	0.94 ± 0.26	1.07 ± 0.50	1.24 ± 0.47	0.095	<0.001
After 1 year (49/39/47)	0.93 ± 0.28	0.88 ± 0.52	1.23 ± 0.41	0.637	<0.001
After 2 years (6/4/16)	1.08 ± 0.20	0.69 ± 0.55	1.28 ± 0.45	0.252	0.313
Methyl-prednisolone doses (mg/day)					
Pre KT (72/67/87)	16.00 ± 0.00	16.84 ± 6.01	15.91 ± 0.86	0.259	0.365
After 1 month (72/65/87)	11.11 ± 1.68	11.97 ± 2.31	11.59 ± 2.70	0.015	0.195
After 3 months (71/55/86)	8.03 ± 1.49	9.09 ± 1.48	8.16 ± 2.06	<0.001	0.637
After 6 months (71/48/71)	6.25 ± 1.82	7.33 ± 1.39	6.87 ± 3.59	0.001	0.197
After 1 year (49/39/48)	4.61 ± 1.48	5.33 ± 1.80	5.96 ± 8.71	0.042	0.289
After 2 years (6/4/16)	3.00 ± 2.45	4.00 ± 0.00	3.75 ± 1.44	0.447	0.505

*Abbreviations*: *KT* kidney transplantation, *MMF* mycophenolate mofetil

^a^group 1 vs. group 2

^b^group 1 vs. group 3

### Incidence of infection

The incidence of infection was lower in group 1 than in the other groups (Table 3). Although the incidence of each infection (CMV, BKV infection, urinary tract infection, pneumonia and sepsis) was not significantly different between groups 1 and 3, the total incidence was significantly lower in group 1. In addition, group 1 showed significantly lower incidence of CMV infection compared with group 2 (2.8 vs. 16.4 %, *p* = 0.007). Pneumonia and sepsis also showed trends of lower incidence in group 1 than in group 2 (1.4 vs. 9.0 %, *p* = 0.056 and 0 vs. 6.0 %, *p* = 0.051, respectively).

**Table 3:** Incidence of infection

	Low dose MMF	Conventional dose MMF		p1^a^	p2^b^
	Rituximab(+)	Rituximab(+)	Rituximab(−)		
	Group 1 (*n* = 72)	Group 2 (*n* = 67)	Group 3 (*n* = 87)		
Incidence of infection	12 (16.7 %)	25 (37.3 %)	30 (34.5 %)	0.007	0.012
Cytomegalovirus	2 (2.8 %)	11 (16.4 %)	5 (5.7 %)	0.007	0.458
BK virus	6 (8.3 %)	9 (13.4 %)	11 (12.6 %)	0.416	0.447
Urinary tract infection	5 (6.9 %)	6 (9.0 %)	14 (16.1 %)	0.758	0.090
Pneumonia	1 (1.4 %)	6 (9.0 %)	4 (4.6 %)	0.056	0.378
Sepsis	0 (0.0 %)	4 (6.0 %)	2 (2.3 %)	0.051	0.501

*Abbreviations*: *MMF* mycophenolate mofetil

^a^group 1 vs. group 2

^b^group 1 vs. group 3

### Graft rejection and serum creatinine levels

Acute cellular rejection occurred significantly more often in group 3 than in group 1 (10.3 vs. 1.4 %, *p* = 0.023). There were no significant differences in other types of graft rejection between groups 1 and 3 (Table 4). Hyperacute rejection did not occur in any group. Chronic rejection occurred in a group 3 patient (0.6 %). There were 2 cases (2.8 %) of antibody-mediated rejection in group 1. However, there were no significant differences in rejection rates between groups 1 and 2. Serum creatinine levels are summarized in Table 5. Although there was no significant difference between groups 1 and 3, serum creatinine levels were lower in group 1 compared with group 2.

**Table 4:** Graft rejection

	Low dose MMF	Conventional dose MMF		p1^a^	p2^b^
	Rituximab(+)	Rituximab(+)	Rituximab(−)		
	Group 1 (*n* = 72)	Group 2 (*n* = 67)	Group 3 (*n* = 87)		
Hyperacute rejection	0 (0 %)	0 (0 %)	0 (0 %)	-	-
Acute cellular rejection	1 (1.4 %)	3 (4.5 %)	9 (10.3 %)	0.352	0.023
Antibody-mediated rejection	2 (2.8 %)	0 (0 %)	0 (0 %)	0.497	0.203
Chronic rejection	0 (0 %)	0 (0 %)	1 (0.6 %)	-	1.000

*Abbreviations*: *MMF* mycophenolate mofetil

^a^group 1 vs. group 2

^b^group 1 vs. group 3

**Table 5:** Serum creatinine levels (mg/dL)

	Low dose MMF	Conventional dose MMF		p1^a^	p2^b^
	Rituximab(+)	Rituximab(+)	Rituximab(−)		
	Group 1 (*n* = 72)	Group 2 (*n* = 67)	Group 3 (*n* = 87)		
Pre transplantation (72/67/87)	8.07 ± 2.82	8.51 ± 2.91	8.72 ± 3.58	0.371	0.213
After 1 month (72/66/87)	1.03 ± 0.60	1.21 ± 1.34	1.03 ± 0.33	0.313	0.930
After 3 months (71/55/86)	1.04 ± 0.26	1.17 ± 0.29	1.11 ± 0.30	0.012	0.153
After 6 months (71/48/71)	1.08 ± 0.27	1.21 ± 0.31	1.14 ± 0.31	0.012	0.181
After 1 year (49/39/48)	1.02 ± 0.26	1.16 ± 0.32	1.07 ± 0.29	0.033	0.388
After 2 years (6/4/15)	1.11 ± 0.20	0.98 ± 0.21	1.35 ± 0.67	0.338	0.397

*Abbreviations*: *MMF* mycophenolate mofetil

^a^group 1 vs. group 2

^b^group 1 vs. group 3

### Incidence of malignancy and mortality

**Fig. 2:**
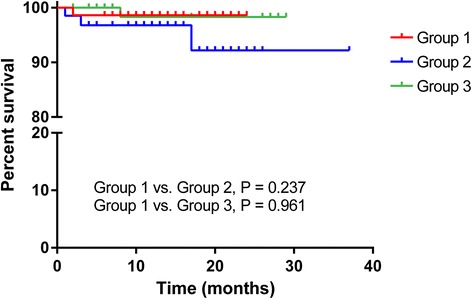
Mortality. One patient in group 1 and one patient in group 3 died due to infectious complications. In addition, 3 patients died in group 2. The cause of death was uncontrolled infection in 2 of them

Malignancy occurred in 2 patients (3.0 %) in group 2 and 1 patient (1.1 %) in group 3, while there were no cases of malignancy in group 1 (Table 6). There was no significant difference in malignancy incidence between the groups. In group 2, malignancies were skin squamous carcinoma and parathyroid cancer. In group 3, one patient was diagnosed with colon cancer at 23 months post-operation. One patient (1.4 %) in group 1 died due to pneumonia aggravation, while there were 3 deaths (4.5 %) in group 2 (Fig. 2) caused by septic shock associated with urinary sepsis, uncontrollable fungal infective endocarditis and metabolic acidosis of unknown origin. One death (1.1 %) caused by pneumocystis pneumonia combined with bacterial infection was reported in group 3. Graft failure occurred in 1 patient in group 2 due to unexpected vessel kinking after the operation.

**Table 6:** Mortality and malignancy

	Low dose MMF	Conventional dose MMF		p1^a^	p2^b^
	Rituximab(+)	Rituximab(+)	Rituximab(−)		
	Group 1(*n* = 72)	Group 2 (*n* = 67)	Group 3 (*n* = 87)		
Mortality	1 (1.4 %)	3 (4.5 %)	1 (1.1 %)	0.237	0.961
Graft failure	0 (0 %)	1 (1.5 %)	0 (0 %)	0.482	-
Malignancy	0 (0 %)	2 (3.0 %)	1 (1.1 %)	0.231	1.000

*Abbreviations*: *MMF* mycophenolate mofetil

^a^group 1 vs. group 2

^b^group 1 vs. group 3

## Discussion

Our present study findings suggest that lower doses of MMF can be safely used in rituximab-treated KT. There was no increase in rejection in patients who used low MMF doses, and infectious complications occurred less frequently.

Cyclosporine, azathioprine and corticosteroid used to be the maintenance immunosuppressants. However, most groups now use tacrolimus, MMF and corticosteroid. More intense immunosuppression has increased the concern about infections.

There have been various trials to reduce immunosuppressant levels. Typically, a dose of 375 mg/m^2^ or 500 mg/body rituximab is used in ABO incompatible or HLA sensitized KT. However, good results have been reported with lower doses of rituximab in ABOi KT. Hiroki et al. [10] reduced the dose of rituximab from 500 mg/body to 200 mg/body and compared clinical outcomes and peripheral CD19 levels. Effective elimination of peripheral blood CD19 cells was observed until 24 months after treatment. In addition, there were no differences in creatinine levels, graft loss, or CMV infection between the groups. Another study compared the outcomes of ABO compatible and ABOi KT using low doses of rituximab (100 mg/m^2^) [11]. Estimated glomerular filtration rate, biopsy-confirmed rejection episodes, acute antibody-mediated rejection, and viral infection did not differ between the groups. The 5-year patient survival rate was 100 % in both groups, while 5-year graft survival rates were 95 % for ABO compatible and 100 % for ABOi transplants (*p* = 0.527). Some studies have even reported ABOi KT without using rituximab [12, 13].

There have been trials to minimize corticosteroid exposure. Kato et al. [14] reported results of an early steroid-withdrawal protocol in 130 patients using cyclosporine, MMF and methyl-prednisolone with basiliximab. The methyl-prednisolone was rapidly tapered and withdrawn on post-transplant day 14. The success rate of steroid withdrawal 12 months after transplantation in recipients of ABOi was 44 %. Galliford et al. [15] performed KT on 10 ABOi patients using 1 week of a steroids protocol with tacrolimus and MMF. Patient- and allograft-survival were 100 % at 1-year post-transplantation in that study. Three patients experienced antibody-mediated rejection within 2 weeks of transplantation, although they were treated successfully. Oettl et al. [16] evaluated late steroid withdrawal after ABOi KT in 15 patients by performing protocol biopsies after 12 to 14 months. If the biopsy did not show signs of rejection, steroid was tapered and eventually stopped after 8 to 12 weeks. However, late steroid withdrawal was successfully performed in only 5 of 11 patients. The remaining 6 patients showed signs of mild acute rejection shortly after complete withdrawal or during steroid tapering.

Our center adopted a low dose of rituximab (200 mg/body) for ABOi KT at the end of 2009. The safety of the early steroid withdrawal protocol was left unproven, and it has been difficult to find data regarding the effectiveness of reduced maintenance immunosuppressant doses. However, in our previous study, we observed that reduced doses of MMF in rituximab-treated KT was safe [8]. CMV infection and pneumonia occurred more often in rituximab-treated KT. For management of infection, the dose of MMF was reduced, but it did not increase the incidence of acute rejection, and graft survival was 100 %. On the basis of this study, we changed our protocol. Our new protocol with lower dose of MMF showed good graft function and reduced incidence of infectious complications. It is a great burden to maintain long-term immunosuppressant treatment with resultant infectious risks in transplanted patients. Therefore, efforts to minimize immunosuppressant levels are justified and will be continued.

Our present study had some limitations. All of our study patients were Korean, and differences in physical and genetic factors between various ethnic groups might affect the clinical results of our protocol for rituximab- and IL-2 receptor antibody- treated KT patients. In addition, the follow-up period was short, necessitating a longer-term follow-up study in the future for decisive clinical decision-making.

## Conclusions

A protocol based on low doses of MMF decreases the infection rate without increasing the incidence of rejection or graft loss. This protocol might be useful for rituximab- and IL-2 receptor antibody-treated KT, although a long-term clinical study is necessary to validate these findings.

## Abbreviations

ABOi: ABO incompatible
CMV: Cytomegalovirus
HLA: Human leukocyte antigen
KT: Kidney transplantation
MMF: Mycophenolate mofetil
